# Alteration of muscle activity during voluntary rehabilitation training with single-joint Hybrid Assistive Limb (HAL) in patients with shoulder elevation dysfunction from cervical origin

**DOI:** 10.3389/fnins.2022.817659

**Published:** 2022-11-09

**Authors:** Margaux Noémie Lafitte, Hideki Kadone, Shigeki Kubota, Yukiyo Shimizu, Chun Kwang Tan, Masao Koda, Yasushi Hada, Yoshiyuki Sankai, Kenji Suzuki, Masashi Yamazaki

**Affiliations:** ^1^School of Integrative and Global Majors, University of Tsukuba, Tsukuba, Japan; ^2^Artificial Intelligence Laboratory, University of Tsukuba, Tsukuba, Japan; ^3^Center for Cybernics Research, University of Tsukuba, Tsukuba, Japan; ^4^Department of Orthopaedic Surgery, Faculty of Medicine, University of Tsukuba, Tsukuba, Japan; ^5^Department of Rehabilitation Medicine, University of Tsukuba Hospital, Tsukuba, Japan

**Keywords:** muscle activity, muscle coordination, Hybrid Assistive Limb (HAL), shoulder rehabilitation, robot-assisted therapy, C5 palsy, compensatory motion, deltoid weakness

## Abstract

Shoulder elevation, defined here as arm raising, being essential for activities of daily living, dysfunctions represent a substantial burden in patients’ lives. Owing to the complexity of the shoulder joint, the tightly coordinated muscular activity is a fundamental component, and neuromuscular impairments have devastating effects. A single-joint shoulder type version of the Hybrid Assistive Limb (HAL) allowing motion assistance based on the intention of the user via myoelectric activation has recently been developed, and its safety was demonstrated for shoulder rehabilitation. Yet, little is known about the physiological effects of the device. This study aims to monitor the changes in muscle activity and motion during shoulder HAL rehabilitation in several patients suffering from shoulder elevation dysfunction from cervical radicular origin. 8 patients (6 males, 2 females, mean age 62.4 ± 9.3 years old) with weakness of the deltoid muscle resulting from a damage to the C5 nerve root underwent HAL-assisted rehabilitation. We combined surface electromyography and three-dimensional motion capture to record muscular activity and kinematics. All participants showed functional recovery, with improvements in their Manual Muscle Testing (MMT) scores and range of motion (ROM). During training, HAL decreased the activity of deltoid and trapezius, significantly more for the latter, as well as the coactivation of both muscles. We also report a reduction of the characteristic shrugging compensatory motion which is an obstacle to functional recovery. This reduction was notably demonstrated by a stronger reliance on the deltoid rather than the trapezius, indicating a muscle coordination tending toward a pattern similar to healthy individuals. Altogether, the results of the evaluation of motion and muscular changes hint toward a functional recovery in acute, and chronic shoulder impairments from cervical radicular origin following shoulder HAL rehabilitation training and provide information on the physiological effect of the device.

## Introduction

Shoulder dysfunctions often have a very detrimental impact on everyday life as upper-limb lifting is essential for activities of daily living. The shoulder complex is a convoluted agglomerate of four different and multiaxial joints (glenohumeral, acromioclavicular, sternoclavicular, and scapulothoracic) allowing a remarkably wide ROM. Shoulder motion relies on complex dynamic interactions between its muscular, osseous, and ligamentous components and heavily depends on tightly coordinated muscle activity (Hawkes et al., 2019). As a result of this dependence, muscular dysfunctions represent a major cause of shoulder motion impairment. Whereas the deltoid muscle is especially fundamental for arm elevation, its weakness can result from neural pathologies affecting the C5 level, notably. Indeed, due to its anatomy, C5 nerve root appears to be particularly vulnerable to damage (Choi et al., 2013). A well-known example might be the C5 nerve root paralysis, or C5 palsy, a serious postoperative complication following cervical spinal surgery. If its exact causes remain mostly obscure, it is widely considered to be caused by an injury of the C5 nerve root during surgery (Joaquim et al., 2019). It can appear after decompression or laminoplasty interventions treating various pathologies such as ossifications of the posterior longitudinal ligament or myelopathies (Joaquim et al., 2019) with an incidence reaching up to 30% in some cases (Ragel and Raslan, 2012). C5 palsy is mainly characterized by muscular weakness in biceps and deltoid, resulting in difficulties in upper limbs elevation. In most cases, the deficits spontaneously resolve after a few months, but some patients never fully recover, and a worsening can even be witnessed. It is thus a very detrimental condition for everyday life (Chang et al., 2013; Miller et al., 2014). Degenerative or postoperative compression or mechanical damage to the C5 nerve root, such as cervical tumor removal or disc herniation, have been reported to lead to very similar symptoms (Chang et al., 2003; Ohnishi et al., 2015). Classic treatment for such impairments comprises electric stimulation, surgical intervention, conservative treatment of steroids, analgesics, and physiotherapy (Shinomiya et al., 1994; Sakaura et al., 2003; Abe et al., 2013). Although a rapid implementation of rehabilitation and intense and repetitive training appear to be crucial factors for recovery (Sicuri et al., 2014), shoulder rehabilitation for elevation dysfunction from neural origin is particularly complex, and the efficiency of those treatments remains too variable. Efficient rehabilitation methods are still lacking.

Over the last decades, robotic devices have been developed and increasingly used as rehabilitation tools. Offering highly repetitive and consistent training, they appear to be particularly appropriate for motor rehabilitation (Turner et al., 2013). Recent studies reported the efficiency of the Hybrid Assistive Limb (HAL, Cyberdyne Inc., Tsukuba, Japan) as a rehabilitation tool for a wide range of motor impairment pathologies (Wall et al., 2015; Endo et al., 2018; Jansen et al., 2018; Saita et al., 2018; Kubota et al., 2019; Ezaki et al., 2021). This intention-based wearable exoskeleton allows the detection of bioelectric signals from still (even slightly) active muscles through surface electrodes and translates these detected signals into movement via actuators and joint motors. It assists the user’s movement in real time, on demand, through a voluntary activation mechanism. In this manner, the HAL exoskeleton integrates neurobiological activity through detection of myoelectric signals reflecting the intention of the user and thus allows a physiological, precise, and efficient rehabilitation. The “Interactive Biofeedback” hypothesis (Morishita and Inoue, 2016; Sankai and Sakurai, 2018) suggests that the coherent sensory feedback resulting from the volitional motion stimulates the impaired neural circuitry and hence enhances neural plasticity and recovery.

To adapt to different pathologies, different types of HAL have been developed. If the most well-known version is the double lower limbs type, single-joint versions adapted to upper limbs have been manufactured more recently (Shimizu et al., 2017, 2019), widening the range of pathology the exoskeleton can operate on. Clinical and motor performances of spinal cord injury and brachial plexus injury cases have been reported to improve after rehabilitation training with these devices. Kubota et al. (2018) reported the safety and efficiency of single-joint HAL on elbow flexion and extension rehabilitation for patients suffering from C5 palsy. However, if upper limb robot-assisted rehabilitation has gained interest these past few years, still remarkably few projects study shoulder training and shoulder robotic assisted rehabilitation.

Makihara et al. (2017) recently demonstrated the feasibility, validity, and safety of shoulder motion assistance using the single-joint HAL in healthy participants. Kubota et al. (2021, 2022) later reported its efficiency for a spinal cord injury case and a C5 palsy case, revealing a potential diminution of compensatory motion. Yet, to the best of our knowledge, such case reports are the only publicly available data, and no reports of other pathologies let alone compilation of several patients have been published.

We aimed here to publish the first study reporting shoulder type HAL training in several patients suffering from cervical pathologies affecting the C5 nerve root and sharing the same symptomatic manifestation, namely a weakness in deltoid muscular activity causing an impairment in humeral elevation, hereafter referred to as “elevation” or “shoulder elevation.” We used a unique combination of electromyography and three-dimensional motion analysis to extensively describe the motion changes and progression of 8 bilateral, unilateral, acute, and chronic shoulder impairment cases undergoing single-joint shoulder HAL training and the underlying physiological components.

## Materials and methods

### Participants

Eight participants (six males, two females, mean age 62.4 ± 9.3 years old) were enrolled in this study. Specific characteristics are detailed in Table 1. All were suffering from shoulder elevation impairments resulting from a damage to the C5 nerve root. Inclusion criteria were weakness of the deltoid but no apparent defect in other shoulder muscles, stable mental state, and no other debilitating condition. The impairments were affecting the right arm for three patients, the left arm for four patients, and both arms for one patient. Among our participants, one was in a chronic state (235 days after symptoms onset). One of our participants was suffering from postoperative palsy due to injury of the C5 nerve root after epidural tumor removal, one from cervical hernia compressing the C5 nerve root and the other six patients were affected by postoperative C5 palsy after surgery for Ossification of Posterior Longitudinal Ligament or Myelopathy. Besides the patient with hernia, none of our patients had any motor deficits of the shoulder before surgery.

**TABLE 1:** Participants’ information.

Patient	Sex	Age	Pathology	Affected arm	HAL start after onset
1	F	64	Postoperative C5 palsy after cervical spondylotic myelopathy	Right	1 day
2	M	62	Postoperative C5 palsy after Ossification of Posterior Longitudinal Ligament	Left	235 days
3	M	58	Postoperative C5 palsy after Ossification of Posterior Longitudinal Ligament	Both	5 days
4	M	62	Postoperative C5 palsy after cervical spondylotic myelopathy	Left	3 days
5	M	76	Postoperative C5 palsy after cervical spondylotic myelopathy	Left	11 days
6	M	73	Postoperative C5 palsy after Ossification of Posterior Longitudinal Ligament	Right	18 days
7	M	58	C5–C6 level radiculopathy due to a herniated cervical disc	Right	71 days
8	F	46	Postoperative palsy after C5 nerve root damage during epidural tumor removal	Left	18 days

As a mean of comparison, data from 12 healthy participants (mean age 29.0 ± 5.2) was recorded during a single session, in identical conditions as for the patients. Mean age of the two groups significantly differed (*P* < 0.0001), but considering the simplicity of the task and on the basis of a previous work stating no difference of scapular muscle activity and balance of activity between different age groups (Morise et al., 2017), we considered that comparison was possible and appropriate.

This study was conducted in accordance with the Declaration of Helsinki and with the approval of the University of Tsukuba Hospital Ethics Committee. All participants gave written informed consent for participation and publication.

### Hybrid Assistive Limb configuration

**FIGURE 1:**
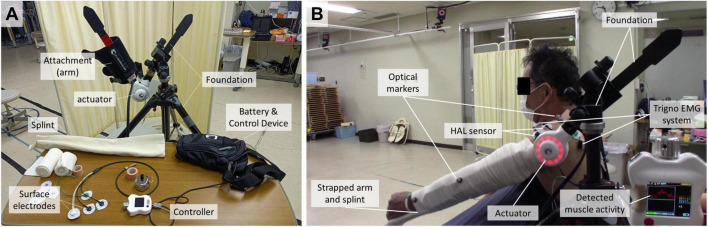
Single-joint type HAL. **(A)** Apparatus set up. **(B)** Fixation for shoulder rehabilitation on the user.

A single-joint HAL adapted to the shoulder motion as described previously (Makihara et al., 2017) was used in this study (Figure 1). The actuator of the device was located right below the acromion so that the HAL was aligned with the shoulder joint. In order to generate appropriate shoulder motion, surface electrodes of the device were positioned on the participant’s middle/anterior fibers of the deltoid to detect bioelectric signals triggering elevation, that is to say arm raising. Gain parameters, which translate muscular activity into torque of the actuator, were adjusted at the start of every session to provide the most comfortable control to the user. Patients were in a sitting position and the HAL was attached in a way that elevation could occur in the scapular plane. This plane, parallel to the scapular body and defined as 30–45° from the frontal plane, was chosen as it minimizes scapular movement and the risk of joint and surrounding structures’ injuries. A custom-made splint was used to keep the patients’ elbow in complete extension.

### Hybrid Assistive Limb intervention

Every session lasted for approximately 1 h, including rest between trials, and started and ended with shoulder elevation exercises as a performance evaluation before and after HAL intervention. Two therapists helped attach and detach the device, a 5-min-long process, and an engineer supervised the data recording. The HAL training intervention itself consisted of 15–450 repetitions of scapular plane shoulder elevation in consideration with fatigue, motivation, and pain of the patient. Rehabilitation was considered completed when patients were able to maintain a voluntary shoulder elevation ROM greater than or equal to 90 degrees for several sessions in a row and presented a stable progression of Manual Muscle Testing (MMT) scores (at least one grade).

### Data collection and analysis

During each session, muscular activity of shoulder muscles (upper fibers of deltoid and trapezius) was recorded via the Trigo™ Lab Wireless Surface EMG system (Delsys Inc., Boston, MA, USA) at 2,000 Hz. Deltoid and trapezius were chosen as the focus of the study. Indeed, deltoid, as the main and virtually only muscle affected by weakness among our patients, was the targeted muscle for recovery. The trapezius was the main contributor to the compensatory motion developed as a response to deltoid weakness. Activity of the infraspinatus (ISP), one of the rotator cuff muscles believed to stabilize the shoulder to achieve elevation (McMahon et al., 1995; Wickham et al., 2010) was also recorded. Since no obvious evolution nor finding enabling any conclusion was identified, it was considered irrelevant to the present study and was briefly presented in Supplementary Figure 1, but not discussed. Kinematics were simultaneously recorded using a three-dimensional motion capture system (16 T20S cameras Vicon MX, Vicon Motion Systems Ltd., Oxford, UK) at 100 Hz. Optical markers of interest were positioned on the lateral epicondyle of the elbow and on the acromion. Marker labeling was carried out using Vicon Nexus software 2.2.3.

Data processing was performed with custom scripts on MATLAB 9.9 2020b (Mathworks, Natick, MA, USA). For each session, 5 cycles of shoulder elevation were detected and averaged from each HAL and no HAL trials using lower peaks of upper arm elevation angle. Dynamic Range of Motion (ROM) of the shoulder elevation angle and of the vertical elevation of acromion were calculated from displacement of the optical markers. EMG activity was band-pass filtered (30–400 HZ), rectified, smoothed by a moving average window filter (50 ms), and then segmented into cycles. Cycle duration was normalized to 0–100% and activity was normalized by maximum muscular activation.

A coactivation index (CAI) of deltoid and trapezius was calculated using a time-dependent co-activation method described in previous works (Rudolph et al., 2000; Ranavolo et al., 2015) (T = number of samples, t = normalized time).


$$CAI=\frac{1}{T}\sum_{t=1}^{T}\left(\frac{\min\left[E\,M\,G\,{\left(t\right)}_{d\,e\,l\,t},E\,M\,G\,{\left(t\right)}_{t\,r\,a\,p}\right]}{\max\left[E\,M\,G\,{\left(t\right)}_{d\,e\,l\,t},E\,M\,G\,{\left(t\right)}_{t\,r\,a\,p}\right]}\right)\times$$

$$\frac{\left(E\,M\,G\,{\left(t\right)}_{d\,e\,l\,t}+E\,M\,G\,{\left(t\right)}_{t\,r\,a\,p}\right)}{2}$$

To evaluate the immediate effect of using HAL on the utilization of muscles in each single session, an adjustment ratio (AR) of deltoid and trapezius muscles, representing the relative reliance on each muscle, was calculated as the ratio of the quotient of the integral value of deltoid activation with HAL/without HAL and the quotient of the integral value of trapezius activation with HAL/without HAL.


$$A\,R=\frac{E\,M\,G_{d\,e\,l\,t}\,H\,A\,L÷E\,M\,G_{d\,e\,l\,t}\,n\,o\,H\,A\,L}{E\,M\,G_{t\,r\,a\,p}\,H\,A\,L÷E\,M\,G_{t\,r\,a\,p}\,n\,o\,H\,A\,L}$$

MMT scores of the deltoid were measured at the beginning of each session.

### Statistical analysis

Statistical analysis was performed with GraphPad Prism version 9.3.1 (GraphPad Software, San Diego, California USA). Paired *t*-tests were used to evaluate differences in ROM, MMT, muscular activity, CAIs, ARs, and acromion elevation. Unpaired *t*-tests were used when comparing with healthy individuals (graphically displayed in gray). Linear regressions were performed on the evolution of ROM, AR, and acromion elevation. Significance will be presented according to standard depiction (ns for *P* > 0.05; * for *P* ≤ 0.05; ^**^ for *P* ≤ 0.01; ^***^ for *P* ≤ 0.001; ^****^ for *P* ≤ 0.0001).

## Results

### Shoulder motion

**FIGURE 2:**
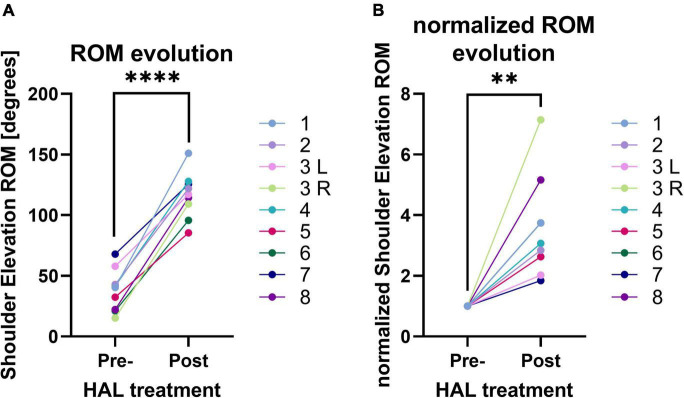
**(A)** Range of Motion (ROM) of shoulder elevation angle before and after HAL treatment. **(B)** Normalized ROM of shoulder elevation angle before and after HAL treatment.

All patients completed sessions without any major adverse effects, and all showed significant progression of motor abilities. The intervention lasted for 14 ± 5 sessions on average. Frequency of the sessions varied, depending on the patients and on discharge, and ranged from 4 sessions per week to 1 session per month. Number of sessions and duration of the intervention are shown in Table 2. Deltoid MMT scores for the first and last session of rehabilitation increased with statistical significance (from 1.56 ± 0.5 to 3.89 ± 0.6; *P* < 0.0001). Passive ROM was already full range before rehabilitation and remained unchanged for all patients except patient 7, for whom it went from 140° before rehabilitation to 170° after, due to pain reduction. Figure 2 presents gross and normalized (from the maximum angle of the first session) dynamic voluntary shoulder elevation angles. ROM significatively increased after HAL treatment (from 38.0° ± 17.4 to 116.5° ± 19.0; *P* < 0.0001). Comparison of the normalized ROM shows a 3.58 fold increase, on average, after rehabilitation (*P* = 0.017). We can notice that the patients with the highest initial ROM (labeled as 7 and 3 L) present the smallest normalized progression while the patients with the lower initial ROM (labeled as 3R and 8) present the highest normalized progression.

**TABLE 2:** MMT scores before and after and details of the HAL intervention.

Patient	1	2	3 L	3 R	4	5	6	7	8
MMT Pre-	2	2	2	2	1	1	1	1	2
MMT Post-	4	4	4	4	4	3	3	5	4
Number of sessions	10	23	7	10	17	18	10	10	18
Time span (weeks)	11	82	2	6	28	42	5	14	23

**FIGURE 3:**
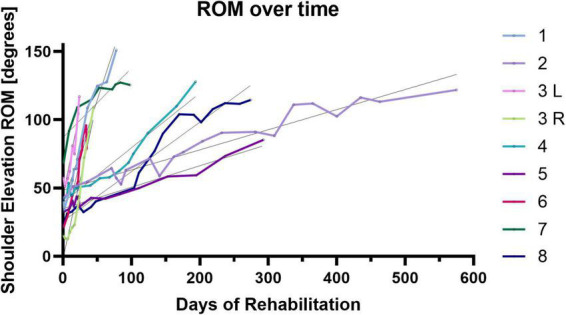
Evolution of ROM of shoulder elevation angle over sessions and linear regression.

Evolution of ROM for all patients and linear regression of curves are presented in Figure 3. Shoulder angle ROM increased over time for all patients. Linear regression analysis demonstrated that slopes were all significantly positive with *P*-values ranging from < 0.0001 to 0.0039 (Table 3). Two clearly distinct progression rate patterns could be noted (linear regression slopes < 0.5 and > 1.5). Slopes were significantly different between the two groups (unpaired *t*-test, *P* < 0.0001) but averaged session frequencies were not (unpaired *t*-test, *P* = 0.2026). Noteworthily, participant 7, who is the cervical hernia patient and had the highest starting ROM, first displayed a steep progression pattern during the first 3 sessions, before displaying a slow progression pattern.

**TABLE 3:** Linear regression analysis of the evolution of range of motion of shoulder elevation angle over time.

Patient	Slope	*R* ^2^	*P*-value	Significance
1	1.524	0.9635	<0.0001	****
2	0.1473	0.9393	<0.0001	****
3 L	2.556	0.8359	0.0039	**
3 R	2.309	0.9323	<0.0001	****
4	0.4009	0.9362	<0.0001	****
5	0.1574	0.9462	<0.0001	****
6	2.113	0.9282	<0.0001	****
7	0.5076	0.7521	0.0012	**
8	0.3629	0.9535	<0.0001	****

### Muscular activity

**FIGURE 4:**
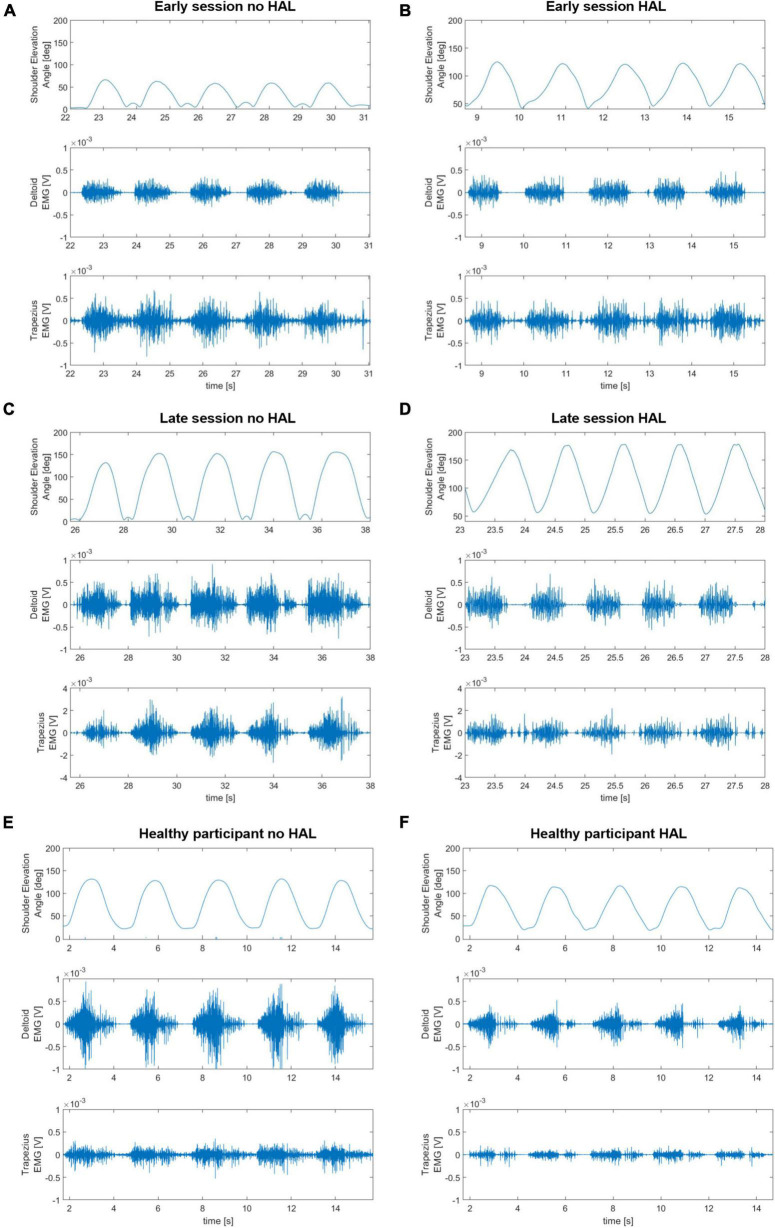
Shoulder elevation angles and surface EMG of deltoid and trapezius without and with the HAL for an early session, on the top **(A,B)**; a late session, in the middle **(C,D)** and a healthy participant, on the bottom **(E,F)**.

Figure 4 shows shoulder elevation angle and surface EMG of deltoid and trapezius for an early and a late session for a representative patient and for a healthy participant. For the patient, a clear augmentation of both muscular activity and ROM can be noticed at the end of rehabilitation. For later sessions, muscular activity, especially from the trapezius, is distinctly more reduced with the HAL exoskeleton. For the healthy participant, without the HAL, the activity of the deltoid is visibly more intense than the activity of the trapezius during arm raising. Both muscles activity is noticeably lower with the HAL.

**FIGURE 5:**
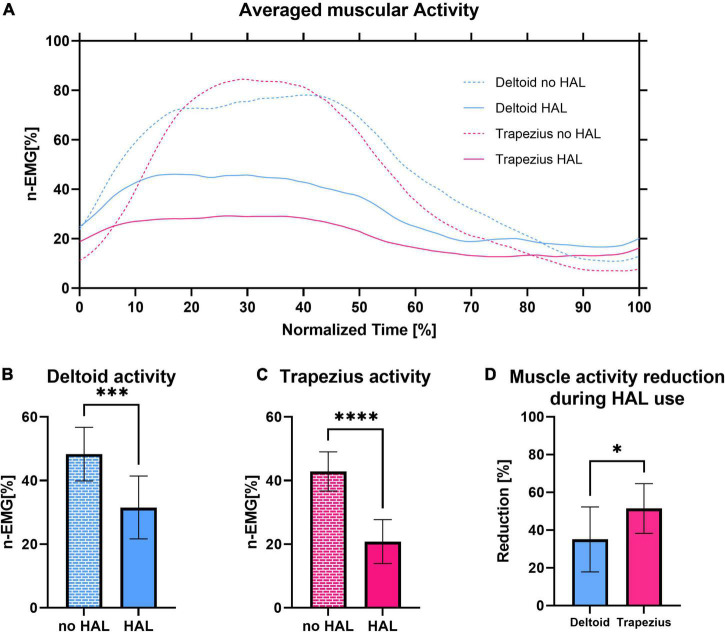
**(A)** Integrated electromyography profiles of deltoid activity without and with shoulder HAL of all patients averaged to a shoulder elevation cycle. **(B)** Normalized activity of the deltoid with and without HAL. **(C)** Normalized activity of the trapezius with and without HAL. **(D)** Comparison of deltoid and trapezius activity reduction during HAL use.

Figure 5 presents electromyography profiles of deltoid and trapezius (panel A) and the reduction of normalized muscular activity (panels B-D) with HAL. During HAL use, activity of both deltoid and trapezius was significantly reduced (48.31 ± 8.42% on average without HAL against 31.54 ± 9.88% with HAL, *P* = 0.0001 for deltoid; 42.86 ± 6.14% on average without HAL against 20.83 ± 6.90% with HAL, *P* < 0.0001 for trapezius). Remarkably, trapezius activity was significantly more reduced than deltoid activity (35.08 ± 17.19% of reduction for deltoid against 51.46 ± 13.15% for trapezius, *P* = 0.0373).

**FIGURE 6:**
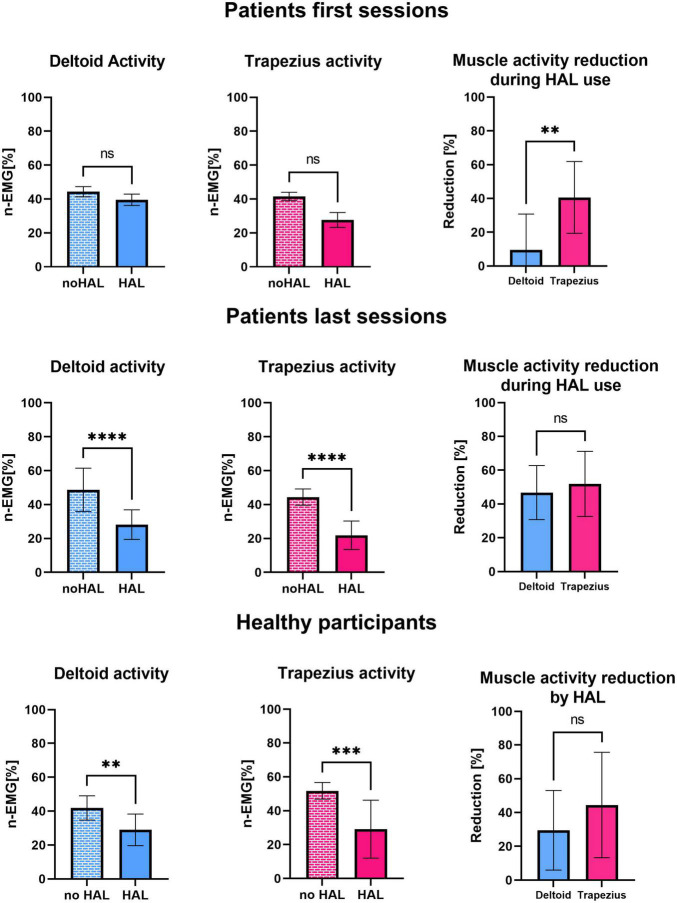
Normalized activity of the deltoid with and without HAL, normalized activity of the trapezius with and without HAL, comparison of deltoid and trapezius activity reduction during HAL use averaged for the first 3 sessions of patients, the last 3 sessions of patients and healthy participants.

To compare the evolution of the effect of HAL on muscular activity at early and late rehabilitation, we averaged the activity of deltoid and trapezius for the 3 first sessions, the 3 last sessions and for healthy participants, as well as the reduction of activity of these muscles during HAL use. The number 3 was chosen in consideration with the number of total sessions as it appeared to be a good compromise to avoid irrelevant inter-session variability while still allowing to see evolution throughout rehabilitation. As shown in Figure 6, during early sessions, activity of the deltoid and the trapezius did not significantly differ with and without the HAL (44.28 ± 3.02% against 39.53 ± 3.31% for deltoid, *P* = 0.1395, and 41.48 ± 2.45% against 27.60 ± 4.43%, *P* = 0.0719; respectively, without HAL and with HAL). Although a reduction of trapezius activity was visible but not significant by itself, it was significantly higher than those of the deltoid (9.63 ± 21.13% against 40.63 ± 21.27%, *P* = 0.0011). In late rehabilitation, both deltoid and trapezius activity are significantly reduced with HAL (48.67 ± 12.77% against 28.18 ± 8.70% for the deltoid, *P* < 0.0001; 44.38 ± 4.80% against 21.87% for the trapezius; respectively, without HAL and with HAL). The percentage of reduction of both muscles did not differ anymore (46.77 ± 15.99% for deltoid against 51.91 ± 19.29% for trapezius, *P* = 0.4651). Similarly, for healthy participants activity of both deltoid and trapezius was reduced with HAL (41.88 ± 7.17% against 28.98 ± 9.34% for deltoid, *P* = 0.0016, and 51.72 ± 4.90% against 29.12 ± 17.04%, *P* = 0.0007; respectively, without HAL and with HAL), and no statistical difference was found in the percentage of reduction of both muscles (29.50 ± 23.56% for deltoid against 43.79 ± 32.28% for trapezius, *P* = 0.0835).

**FIGURE 7:**
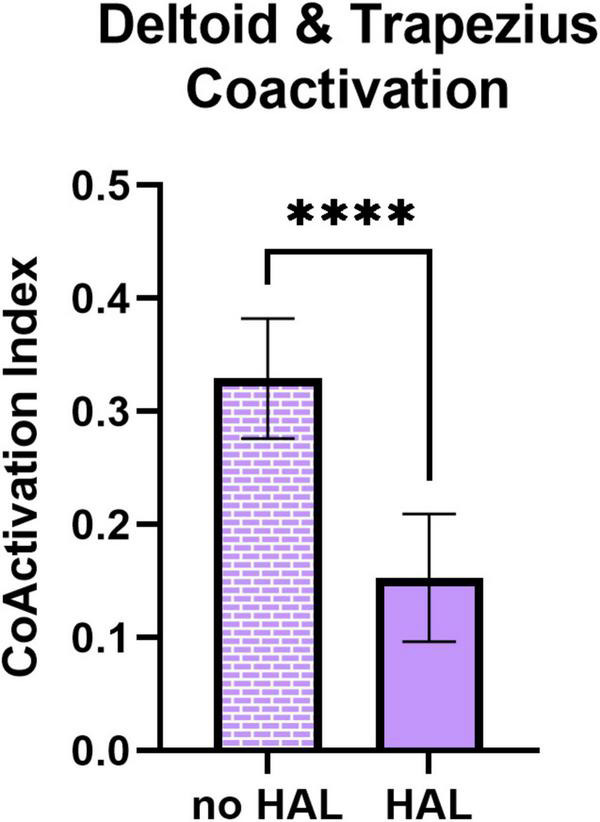
Coactivation index (CAI) of deltoid and trapezius without and with HAL.

Figure 7 presents the time-dependent CAI of deltoid and trapezius. Coactivation of deltoid and trapezius is significantly reduced while using HAL in all patients (0.329 ± 0.05 without HAL against 0.153 ± 0.06 with HAL, *P* < 0.0001).

**FIGURE 8:**
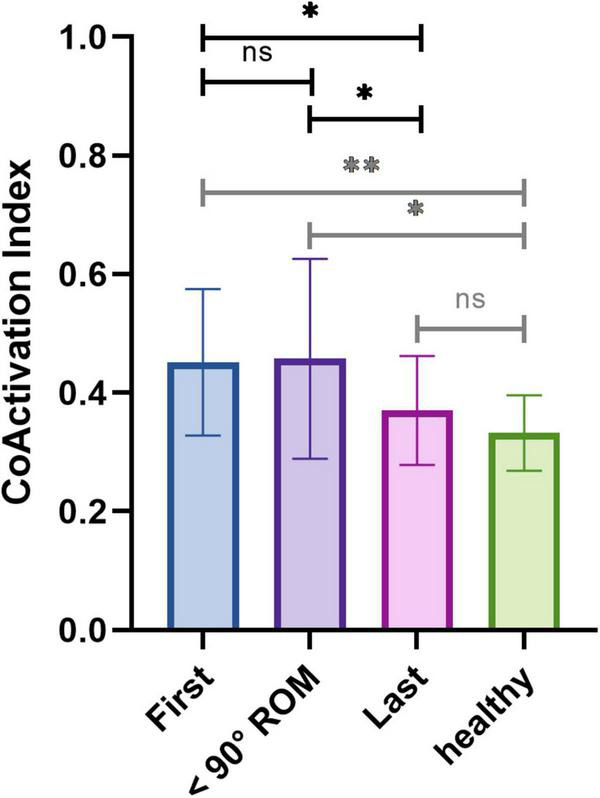
Averaged coactivation index of deltoid and trapezius for the first three sessions, the three sessions preceding the reaching of the 90 degrees ROM and the last three sessions of patients, and of healthy participants.

As the evolution of the coactivation of deltoid and trapezius did not appear to be linear over time, we decided to segment the rehabilitation in 3 phases. We averaged the CAI of the three first sessions, the three sessions preceding the reaching of 90 degrees ROM, and the three last sessions. As shown in Figure 8, if the CAI of the first sessions and the sessions preceding the reaching of 90 degrees are not significantly different from each other (respectively, 0.452 ± 0.123 and 0.457 ± 0.169, *P* = 0.8818), both are significantly higher than the CAI in the last sessions (0.370 ± 0.092, *P* = 0.0433 and *P* = 0.0462, respectively). This difference is all the more substantial considering that the reaching of 90 degrees occurred during the last quarter of rehabilitation (23% before the end, on average). No statistical difference was found between the last session and the data from healthy participants (0.333 ± 0.064, *P* = 0.2784) while CAI from both the first sessions and sessions preceding the reaching of 90 degrees ROM were statistically higher than the data from healthy participants (respectively, *P* = 0.0095 and *P* = 0.0288).

**FIGURE 9:**
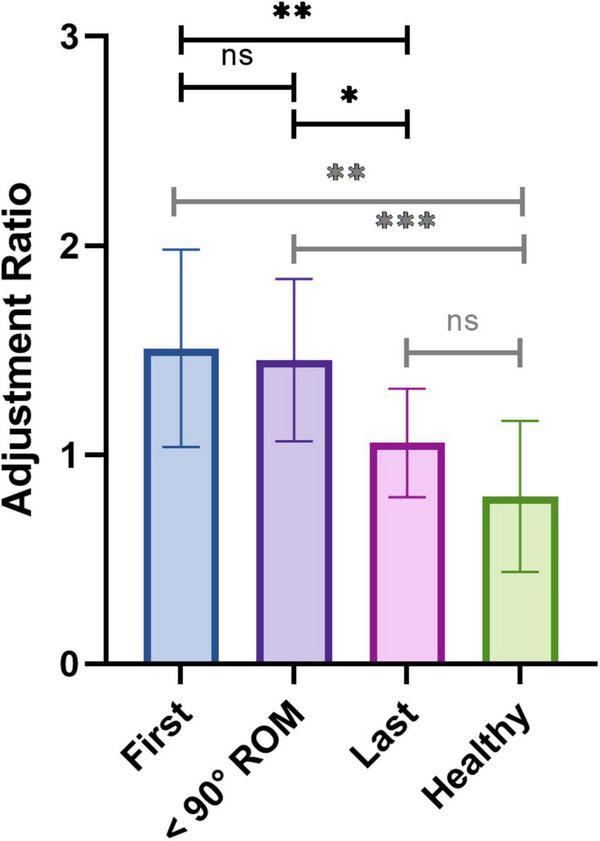
Averaged adjustment ratio for the first three sessions, the three sessions preceding the reaching of the 90 degrees ROM, and the last three sessions of patients, and of healthy controls.

To assess the relative utilization of deltoid and trapezius, we calculated the AR of both muscles as the quotient of the ratio of deltoid activity with HAL over without HAL over the ratio of trapezius activity with HAL over without HAL. This AR diminished over sessions for all patients. Linear regression analysis (Supplementary Table 1) showed that all slopes were negative but were significant for three patients only. It is to be noted that the fitness of the slope was relatively poor, with a mean squared R of 0.26 ± 0.28. We averaged the AR for the first three sessions, the three sessions preceding the reaching of 90 degrees ROM and last three sessions of each patient, similarly to what we did for the CAI. As presented in Figure 9, the AR was significantly smaller at the end of the rehabilitation compared to the beginning (1.508 ± 0.472 at the beginning vs. 1.056 ± 0.260 at the end, *P* = 0.0090) and compared to the sessions preceding the reaching of 90 degrees ROM (1.452 ± 0.388, *P* = 0.0272). No significant difference was observed between the first sessions and the sessions preceding the reaching of 90 degrees ROM (*P* = 0.2223). Similarly, no statistical difference was found between the AR of the last sessions and the AR of healthy participants (0.801 ± 0.361, *P* = 0.1018), while a significant difference was found between healthy participants and the patients’ first sessions and sessions preceding the reaching of 90 degrees ROM (*P* = 0.0013 and *P* = 0.0008, respectively).

### Shrugging motion

**FIGURE 10:**
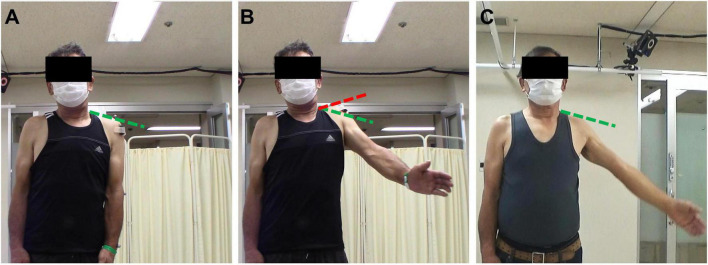
Characteristic shrugging motion observed in patients to compensate for weakness of the deltoid. The green hatched line marks the acromion elevation during a relaxed state of the scapula (A) and is the same for the three pictures. The red hatched line marks the acromion elevation during the excessive shrugging compensatory motion. **(A)** Rest state. **(B)** Compensatory motion (first session, patient 4). **(C)** No compensatory motion (last session, patient 4).

**FIGURE 11:**
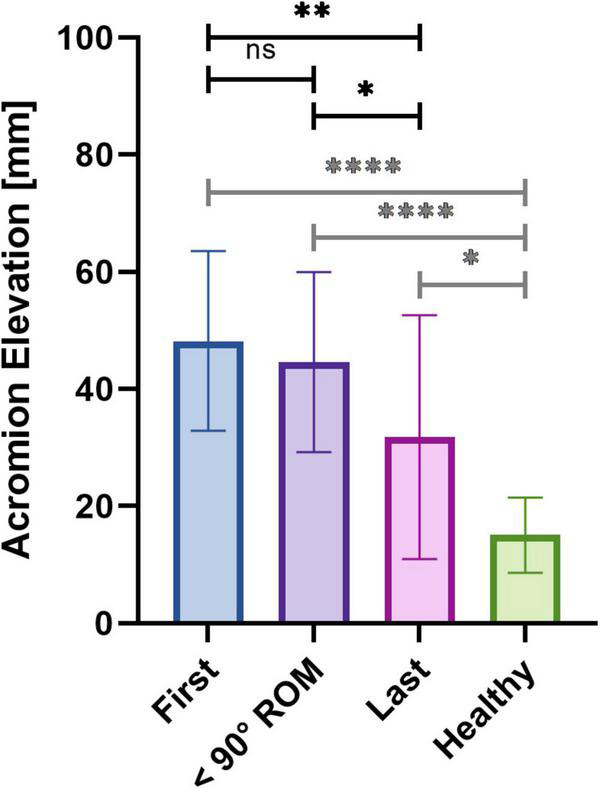
Averaged relative acromion elevation for the first three sessions, the three sessions preceding the reaching of the 90 degrees ROM and the last three sessions of patients, and of healthy controls.

To monitor the evolution of the characteristic compensatory shrugging motion (Figure 10), we evaluated the shoulder elevation motion by monitoring the elevation of the acromion. In order to compensate for the difference of shoulder elevation angle along the rehabilitation process, we examined the elevation of the acromion of the patients for a fixed ROM, being the maximum ROM measured in the first session. Linear regression analysis revealed that the relative acromion elevation diminished throughout the rehabilitation for all patients, although this diminution was statistically significant for four of them only (Supplementary Table 2). Similarly, as presented in Figure 11, the acromion elevation was significantly diminished for the last sessions compared to the first sessions (48.20 ± 15.33 for the first sessions, 31.82 ± 20.82 for the last sessions, *P* = 0.0029), and the sessions preceding the reaching of 90 degrees ROM (44.59 ± 15.40 before 90 degrees, *P* = 0.0113). Again, no significant difference was found between the first sessions and the sessions preceding the reaching of 90 degrees ROM (*P* = 0.2711). Acromion elevation of healthy participants was statistically lower than those of patients (15.05 ± 6.44, *P* < 0.0001 for first sessions, and sessions preceding the reaching of 90 degrees ROM, and *P* = 0.0214 for last sessions).

## Discussion

In the present study, we evaluated the changes in muscle control and motion during rehabilitation training using the shoulder version single-joint HAL suit in patients suffering from shoulder elevation impairments originating from a damage to the C5 nerve root. Our eight participants were suffering from C5 palsy, C5 level hernia and postoperative C5 level paralysis following an epidural spinal cord tumor removal, and all were sharing a common symptomatic manifestation being a weakness of the deltoid. They underwent HAL therapy consisting of repetitive voluntary shoulder elevation in the scapular plane using the HAL exoskeleton. All patients presented motor progression. The ROM of shoulder elevation angle as well as MMT scores significantly increased after HAL treatment, thus indicating functional recovery.

HAL detects bioelectric signals (reflecting muscles action potentials) via surface electrodes located over relevant muscles and amplifies and translates them in real-time into appropriate movement, thus assisting voluntary motion. The amplification allows its utilization with muscular activity as low as MMT 1. The performed movement will be voluntary and in accordance with the intention of the user. Both visual and propriosensory feedback will be generated and such feedback has been shown to trigger neuroplasticity and to be essential in the recovery process (Broderick et al., 2018; Takeoka and Arber, 2019). We suppose that the sensory feedback resulting from physiologically accurate motion in accordance with internal predictions can induce modifications in the central nervous system that will translate into a change in muscle recruitment allowing the functional recovery that was observed. Repetitive training has been reported to be a key factor as well. But more importantly, the HAL allows the adjustment of its assistance of movement. Indeed, gain and torque parameters can be adjusted to fit best the user’s needs all along their recovery process. In addition, gravity compensation has been reported to facilitate motor training in cases of weakness of the upper limb (Kloosterman et al., 2010; Huang et al., 2017). Hence, we hypothesize that the shoulder HAL is particularly appropriate for rehabilitation of dysfunctions from neural origin and has the potential to trigger recovery.

We looked at the effect of HAL on muscular activity. Both deltoid and trapezius muscle displayed a lower activity with the HAL. A reason behind this diminished muscle activation with the HAL can be the reduced arm weight. Indeed, the assistance is such that the muscle activity necessary to move against gravity is lower. Decreased muscle activity, especially of the deltoid, during limb raising with gravity compensation has already been widely documented (Kloosterman et al., 2010). Comparing early to late rehabilitation, we can see that the decrease of muscle activity, especially deltoid activity, with the HAL was not present from the beginning but appeared during later stages of rehabilitation and can be observed in healthy participant as well. It can be interpreted as an increase of non-assisted muscle contraction. Globally, for patients, the activity of trapezius was more reduced than the activity of deltoid during the use of HAL. It can be interpreted as a HAL-induced diminution of tricky shrugging compensatory motion. In the case of deltoid weakness, many patients develop a trapezius-mediated compensatory shrugging motion during shoulder elevation (Figure 10). This trick motion can be an obstacle to recovery as it prevents normal use of the muscles. Indeed, previous studies (Wickham et al., 2010; Hawkes et al., 2019) report that in normal condition, upper trapezius is activated at its maximum around 90–100 degrees during arm raising motion. It is considered that the upper trapezius has a stabilizer and upward rotator role for the scapula and whose contribution in the elevation occurs mainly after 90 degrees of shoulder elevation (Scibek, 2012). Deltoid mainly provides force before the reaching of 90 degrees (McMahon et al., 1995). However, in the case of deltoid weakness, individuals tend to compensate for this lack of strength with a shrugging motion resulting from an excessive activation of upper trapezius (Kubota et al., 2021, 2022). A substantial reduction of trapezius’ use during elevation with the HAL exoskeleton could suggest that the HAL increased reliance on the deltoid, and decreased reliance on the trapezius. This increased reduction of trapezius activity compared to deltoid activity is lesser at the end of the rehabilitation and in healthy participants, suggesting that the excessive trapezius activation is less substantial in those cases.

We found that the coactivation of deltoid and trapezius (represented by the CAI) was significantly reduced when using the HAL exoskeleton. We believe that the single-joint shoulder HAL, with its actuator located on the center of rotation of the shoulder and its fixation, inhibits the motion of the scapula and allows the use of relevant muscles only (in our case, deltoid), thus avoiding this compensatory motion. We presume that this configuration prevents the learning of this pernicious motion in the case of acute patients and diminishes it for chronic ones. Incidentally, it is a possible reason behind the delay of progression in chronic patients: this trick motion, detrimentally learned to compensate for the muscular weakness of deltoid during shoulder elevation, should be discouraged in order to retrieve a closer-to-normal movement.

To monitor its evolution over time, we investigated the changes of coactivation of deltoid and trapezius during unassisted voluntary elevation across rehabilitation. We found a non-linear and belated global diminution of the CAI between the first and last sessions of HAL intervention. Hence, we made the assumption that this diminution of CAI was a second-phase effect of HAL treatment, and decided to segment the rehabilitation process for the analysis around a threshold being the reaching of a 90 degrees ROM. Our results corroborated this segmentation, as we report that the CAI of the three sessions preceding the reaching of a 90 degrees ROM did not significantly differ from the first sessions but was significantly higher than those of the last sessions, thus hinting toward a delayed diminution of compensatory motion. It is noteworthy to mention that this reaching of 90 degrees happened late in the rehabilitation, in the last quarter on average. It thus appears that coactivation stayed stable for most of the rehabilitation before dropping and reaching levels similar to healthy participants during the last sessions. Interestingly, the CAI of the sessions preceding the reaching of 90 degrees ROM was slightly, but not statistically, superior to the CAI of the first sessions. This slight increase, although not significant, could be interpreted as an initial recovery, and thus increase, of deltoid activity. We believe that HAL training, focusing on deltoid, allows the increase of activity of this muscle. Once patients recovered a sufficient deltoid strength, they become able to perform sufficient elevation without any excessive reliance on the trapezius and the compensatory motion thus consequently disappears.

To evaluate the extent of changes in muscle coordination induced by the HAL in each session, we calculated an AR, defined as the ratio of the quotient of the integral value of deltoid activation with HAL/without HAL and the quotient of the integral value of trapezius activation with HAL/without HAL. When greater than one, by definition, this ratio represents a HAL-mediated shifting of the muscular coordination toward an increasing reliance on the deltoid and a decreasing reliance on the trapezius during shoulder elevation, therefore suggesting that the HAL shifts the muscular coordination from a trapezius-lead compensatory motion toward a closer-to normal deltoid-lead shoulder elevation. Our results showed that the AR remained greater than one for early sessions and sessions before reaching 90 degrees ROM (Figure 9), suggesting that the HAL’s adjustment of the muscle coordination was effective during these sessions. Indeed, we suggest that the trapezius activity decreases with the suppression of compensatory motion but will always remain higher without the HAL than while using it, the ratio *EMG*_*trap*_*HAL*÷*EMG*_*trap*_*noHAL* will thus tend to augment without ever reaching one. At the same time, the ratio *EMG*_*delt*_*HAL*÷*EMG*_*delt*_*noHAL* will decrease, as the voluntary contraction of the deltoid without the exoskeleton will increase throughout rehabilitation. The augmentation of deltoid activity and the diminution of excessive trapezius activity will thus translate into a diminution of the AR. Our results demonstrate a global diminution tendency of this ratio over rehabilitation time, tending toward levels found in healthy individuals. This reduction can be interpreted as a supplantation of trapezius activity by deltoid activity during unassisted voluntary motion, indicating once again a diminution of the compensatory motion. Such a tendency has already been shown in a previous work (Kubota et al., 2021) as a linear decay of the AR throughout the sessions. However, in the present study, this decrease was significant for three patients only and the fit of the linear regression analysis we performed was globally poor. When segmenting the different phases of HAL intervention, we noticed that this diminution mainly occurred in the later phase of the rehabilitation, after the reaching of 90 degrees ROM, similarly to our report of deltoid and trapezius coactivation. We interpret this delay as a further hint toward a two-steps effect of shoulder HAL: an early augmentation of deltoid activity, occurring while the patients were repeatedly practicing the adjusted coordination until they assimilated the muscle pattern, followed by a diminution of excessive trapezius-mediated compensatory motion in a later phase, accompanied by a shoulder elevation greater than 90 degrees. In this later phase, the AR tended toward one and toward healthy participants’ level, indicating that the HAL’s effect turned into a simpler power assistance.

To further confirm the putative diminution of compensatory motion hinted at by the diminution of trapezius activity and of AR, we looked at the elevation of the acromion. In normal condition, the initial phase of shoulder elevation is a stabilization phase, the scapula will then mainly contribute to the elevation after 90 degrees (Scibek, 2012). It implies that the acromion will elevate mainly in the later phase of the shoulder elevation process. To bypass the physiological increase of acromion elevation induced by the increase of ROM, and to be able to compare all sessions regardless of the motor performance, we examined the elevation of the acromion of the patients for a fixed ROM, being the maximum ROM measured on the first session. All patients showed a decreasing tendency over time, although linear regression analysis revealed that this tendency was significant for four patients only. Similarly to the CAI and AR, when separating the different phases of HAL intervention, it appeared that the diminution of acromion elevation also mainly occurred in the later phase of rehabilitation, after the reaching of 90 degrees ROM. Again, results at the end of rehabilitation tended toward levels of healthy participants, although still being statistically different. We believe that this difference can result from additional time necessary for some patients to overcome the compensatory motion they have been using since the deltoid impairment appeared. It is worth noting that the variability was the highest for the last sessions. These results support our belief of an action of HAL on the compensatory shrugging motion in the later phase of the rehabilitation.

Taken altogether, the data corroborate the reaching of a 90 degrees ROM as a recovery threshold and suggest a two-steps effect of shoulder HAL. The mechanism we propose is as follows. The recovery of a sufficient deltoid activity is necessary to reach a 90 degrees ROM. Once it is achieved, through repetitive HAL training, the shrugging compensatory motion will no longer be necessary, and will gradually disappear. This effect is illustrated by the correlated decrease of CAI of deltoid and trapezius, AR and acromion elevation after the reaching of 90 degrees ROM. The reliance on deltoid will be increased, diminishing the CAI; the adjustment effect of HAL will cease, which will be translated into the AR tending toward 1; and the shrugging motion will decrease, as shown by the diminution of acromion elevation during the initiation of the shoulder elevation.

Interestingly, two distinct patterns of recovery rate could be noticed from the ROM evolution over time and patients could be divided into “fast learners” (linear regression slope over 1.5) and “slow learners” (linear regression slope under 0.5) with the notable exception of patient 7, who first presented a “fast” learning pattern before quickly shifting to a slower pattern. This shifting of progression could suggest a plateauing of performance, which was not observed in the other patients. The averaged frequency of sessions did not differ between the two groups and no clear difference was found between acute and chronic patients as both types of patients could present slow learning profiles. A previous study reports that different patients are more or less receptive to HAL treatment: Kadone et al. (2020) presented what seems to be a non-responding patient to HAL rehabilitation. These interindividual differences in response to treatment, if not fully understood yet, might result from previous pathological history. Further research with more patients is necessary to clarify this phenomenon, which is crucial to offer a rehabilitation as adapted as possible to each patient.

Additionally, the fact that our participants suffered from different pathologies is not to be neglected. Although all of them presented deltoid weaknesses originating from a damage to the C5 nerve root, both degenerative and postoperative damages were reported. It might thus be possible that the HAL affects the impaired circuitry differently. Yet, no clear difference was found between pathologies in this work. Additionally, we report data from a limited number of patients, and our results cannot be generalized. More extensive study with more patients is, again, needed. In addition to the variability of pathologies, our group of participants presented a substantial variation of training range and frequency. The rehabilitation period ranged from 24 to 575 days (mean = 179.6 ± 179.8) and a significant diminution of frequency occurred after discharge of the patients from the hospital. Yet, we report no decrease of progression rate after the discharge of patients. Moreover, in cases where a long interval occurred between sessions, no regression of ROM was observed, hinting toward a stable recovery effect of HAL. This may be clarified in a future study with longitudinal follow-up and long-term assessments.

The present study did not aim to prove the better efficiency of HAL treatment compared to traditional therapy but to monitor and analyze the evolution of motion and muscle activity of patients during shoulder HAL training. Yet, for comparative purposes, traditional rehabilitation for upper limb elevation weakness from spinal origin mainly consists of physical therapy (Sakaura et al., 2003). Literature reports that patients usually take between 3 and 6 months to recover (Sakaura et al., 2003) and up to 1 year to reach a new functional baseline (Pennington et al., 2020). In some cases, no recovery can be witnessed, and a worsening can even happen (Chang et al., 2013). It is globally accepted that patients with a lower MMT and a longer time after onset, have the lowest recovery rates and the longest times to recover (Imagama et al., 2010; Miller et al., 2014; Chung et al., 2015). Miller et al. (2014) report that patients with severe C5 palsy (with an initial MMT of 2 or less) recover on average 25% less than those with MMT of 3 or 4, and 44% of them take more than 6 months to recover.

In our study, all patients had an initial deltoid MMT of 2 or less and 7 of them were over 50 years old. For the patient suffering from a herniated disc (patient 7), the only one whose damage was degenerative, no motor improvement occurred after surgery and his MMT score stagnated at level 1. Yet, after 98 days of HAL treatment, he reached MMT 5, thus matching successful surgery results previously reported (Chung et al., 2015). Little literature reports cases of severe motor weakness following a C5 nerve root extensive dissection during tumor removal that was not present prior to surgery. Nevertheless, a couple of studies report transitive light palsy in most cases but persisting weakness of the deltoid when postoperative palsy was initially severe (McCormick, 1996; Celli et al., 2005). In comparison, our patient whose nerve root has been injured during resection presented a MMT 2 after surgery and reached MMT 4 after 23 weeks. Our patients suffering from acute C5 palsy recovered and all augmented their MMT by at least 2 grades (reaching MMT 3 or 4) in 4.5 months on average. Moreover, one of our patients (patient 2) was in a chronic state and showed no sign of recovery after traditional physical therapy. This patient successfully recovered a ROM superior to 120 degrees and increased his MMT score in 18 months. We believe that taken altogether, our results suggest the efficiency of shoulder HAL rehabilitation. However, the present results alone do not allow any objective conclusion of a better efficiency than traditional therapy, and further research involving double blind control studies is necessary.

Thus, the present study indicates HAL-assisted rehabilitation as feasible and suitable for recovery of deltoid-related shoulder elevation impairments following damage to the C5 nerve root. It suggests that shoulder HAL training relying on deltoid induces a two-steps effect, namely an increase of the weakened deltoid activity in a first phase, followed by a diminution of pernicious compensatory motion in a second time. As mentioned in the literature (Sicuri et al., 2014), a rapid implementation of repetitive rehabilitation training seems to be a key for efficient recovery. Yet, the present results also report the motor progression of a chronic patient for whom traditional physical therapy was ineffective. More extensive studies with more patients and including randomized controlled trials remain to be done to further validate the effect of shoulder HAL. Furthermore, we describe a quantitative method to evaluate the changes in muscle use and reliance throughout rehabilitation via coactivation measurement and AR. We hope it can provide a basis, a reference for future research in the field of robot-assisted rehabilitation.

## Data availability statement

The datasets presented in this article are not readily available because myoelectric data being considered personal biometric data, a new approval from the University of Tsukuba Hospital Ethics Committee is necessary before making it available. Requests to access the datasets should be directed to ML, margaux@ai.iit.tsukuba.ac.jp.

## Ethics statement

The studies involving human participants were reviewed and approved by the University of Tsukuba Hospital Ethics Committee. The patients/participants provided their written informed consent to participate in this study.

## Author contributions

ML and HK collected, analyzed, and interpreted the data. ML wrote and drafted. HK edited the manuscript. SK administered HAL therapy and collected clinical scores. YSh supported the HAL therapy. CT assisted the data analysis. MK and MY operated the cervical patients. MK and YH provided important comments on the planning and implementation of shoulder HAL treatment. YSa originally developed the robot suit HAL and conceived the idea of HAL therapy. KS provided essential ideas for the analysis. MY developed HAL therapy for the cervical patients and organized the study. All authors made critical revisions of the manuscript and approved the final version.

## Supplementary material

Supplementary Figure 1 Averaged coactivation index **(left)** and adjustment ratio **(right)** of deltoid and infraspinatus for the first three sessions, the three sessions preceding the reaching of the 90 degrees ROM and the last three sessions of patients, and for healthy participants. No difference of the coactivation of deltoid and infraspinatus was found throughout the rehabilitation and when compared with healthy participants’ data. Similarly, we found no statistical difference of the adjustment ratio of deltoid and infraspinatus throughout the rehabilitation and when compared with healthy participants’ data. The visible diminution of AR between the first sessions and three sessions preceding the reaching of 90 degrees ROM, although non-significant, could be due to the increase of deltoid activity. Indeed, this AR was calculated as the ratio of the quotient of the integral value of deltoid activation with HAL/without HAL and the quotient of the integral value of infraspinatus activation with HAL/without HAL. The decrease, being more severe at the beginning of the rehabilitation, strengthens our hypothesis of a 2 steps effect of HAL.
