# Supplementary figures and images for: Alteration of muscle activity during voluntary rehabilitation training with single-joint Hybrid Assistive Limb (HAL) in patients with shoulder elevation dysfunction from cervical origin
Source: Front Neurosci. 2022 Nov 9;16:817659. doi: 10.3389/fnins.2022.817659 (PMC9682184; doi:10.3389/fnins.2022.817659)

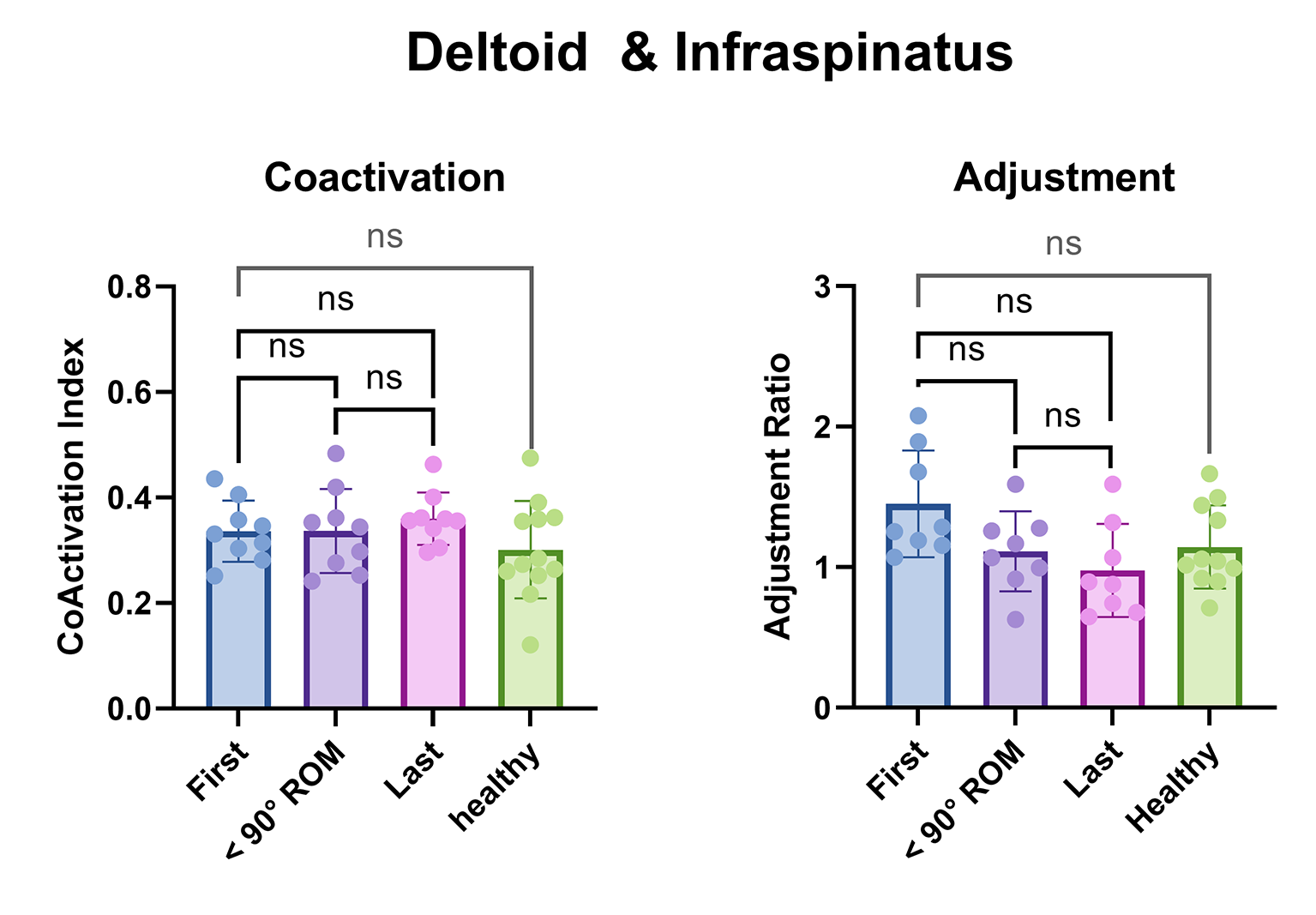

Supplement: Supplementary Figure 1 — Averaged coactivation index (left) and adjustment ratio (right) of deltoid and infraspinatus for the first three sessions, the three sessions preceding the reaching of the 90 degrees ROM and the last three sessions of patients, and for healthy participants. No difference of the coactivation of deltoid and infraspinatus was found throughout the rehabilitation and when compared with healthy participants’ data. Similarly, we found no statistical difference of the adjustment ratio of deltoid and infraspinatus throughout the rehabilitation and when compared with healthy participants’ data. The visible diminution of AR between the first sessions and three sessions preceding the reaching of 90 degrees ROM, although non-significant, could be due to the increase of deltoid activity. Indeed, this AR was calculated as the ratio of the quotient of the integral value of deltoid activation with HAL/without HAL and the quotient of the integral value of infraspinatus activation with HAL/without HAL. The decrease, being more severe at the beginning of the rehabilitation, strengthens our hypothesis of a 2 steps effect of HAL. [file Image_1.TIF]
