# Supplementary material for: Alteration of muscle activity during voluntary rehabilitation training with single-joint Hybrid Assistive Limb (HAL) in patients with shoulder elevation dysfunction from cervical origin
Source: Front Neurosci. 2022 Nov 9;16:817659. doi: 10.3389/fnins.2022.817659 (PMC9682184; doi:10.3389/fnins.2022.817659)
Supplement: Supplementary file 2 [file Table_1.docx]

| Patient | Slope | R^2^ | P-value | Significance |
| --- | --- | --- | --- | --- |
| 1 | -0.01534 | 0.6525 | 0.0047 | ** |
| 2 | -0.0006589 | 0.05856 | 0.2659 | NS |
| 3 L | -0.06689 | 0.7836 | 0.019 | * |
| 3 R | -0.003154 | 0.01068 | 0.7763 | NS |
| 4 | -0.003528 | 0.1173 | 0.1784 | NS |
| 5 | -0.0002057 | 0.003806 | 0.8271 | NS |
| 6 | -0.04968 | 0.3223 | 0.0869 | NS |
| 7 | -0.001915 | 0.1009 | 0.3711 | NS |
| 8 | -0.002489 | 0.2951 | 0.0198 | * |

**Supplementary Table 1:** Linear regression analysis of the adjustment ratio of deltoid over trapezius activity over time
