# Supplementary material for: Alteration of muscle activity during voluntary rehabilitation training with single-joint Hybrid Assistive Limb (HAL) in patients with shoulder elevation dysfunction from cervical origin
Source: Front Neurosci. 2022 Nov 9;16:817659. doi: 10.3389/fnins.2022.817659 (PMC9682184; doi:10.3389/fnins.2022.817659)
Supplement: Supplementary file 3 [file Table_2.docx]

| Patient | Slope | R^2^ | P-value | Significance |
| --- | --- | --- | --- | --- |
| 1 | -0.4333 | 0.8177 | 0.0003 | *** |
| 2 | -0.04648 | 0.3 | 0.0068 | ** |
| 3 L | -0.03164 | 0.0007779 | 0.9527 | NS |
| 3 R | -0.5686 | 0.5053 | 0.0212 | * |
| 4 | -0.08283 | 0.2488 | 0.0415 | * |
| 5 | -0.04518 | 0.1979 | 0.0643 | NS |
| 6 | -1.276 | 0.7541 | 0.0024 | ** |
| 7 | -0.1642 | 0.3274 | 0.0839 | NS |
| 8 | -0.02772 | 0.1174 | 0.1639 | NS |

**Supplementary Table 2:** Linear regression analysis of the elevation of the acromion over time
